# Supplementary material for: Insights into Drug Cardiotoxicity from Biological and Chemical Data: The First Public Classifiers for FDA DICTrank
Source: bioRxiv. 2023 Oct 18:2023.10.15.562398. Preprint. [Version 1] doi: 10.1101/2023.10.15.562398 (PMC10614794; doi:10.1101/2023.10.15.562398)
Supplement: Supplement 1 [file media-1.docx]

SUPPLEMENTARY FIGURES

Insights into Drug Cardiotoxicity from Biological and Chemical Data: The First Public Classifiers for FDA DICTrank

Srijit Seal*^1^, Ola Spjuth^2^, Layla Hosseini-Gerami^3^, Miguel García-Ortegón^4^, Shantanu Singh^1^, Andreas Bender^4^, Anne E. Carpenter^1^

^1^Imaging Platform, Broad Institute of MIT and Harvard, US
^2^Department of Pharmaceutical Biosciences, Uppsala University, Sweden

^3^Ignota Labs, UK

^4^Department of Chemistry, University of Cambridge, UK

Documentation: <https://broad.io/DICTrank_Predictor>

Code: <https://github.com/srijitseal/DICTrank>

Datasets: 10.6084/m9.figshare.24312274

*Correspondence:

[seal@broadinstitute.org](mailto:seal@broadinstitute.org)

**
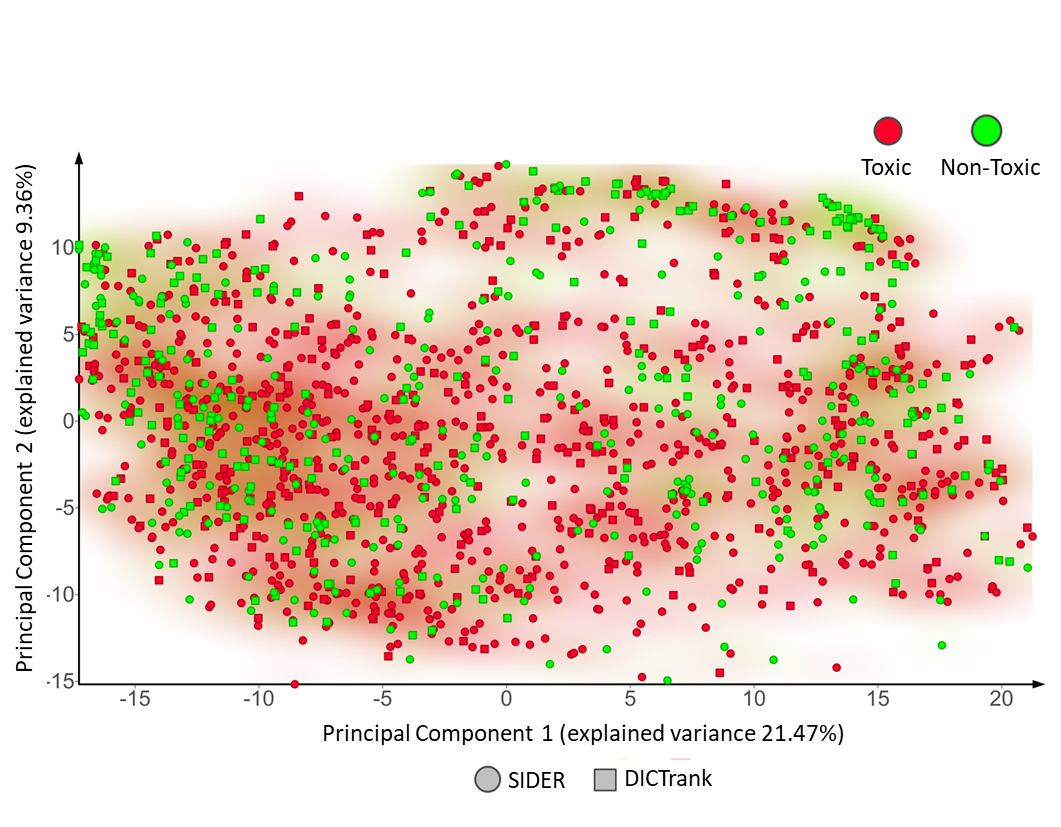
**

Figure S1: The chemical space of SIDER compounds and DICTrank compounds as defined by FragFP fingerprints (chemical fingerprints) in a principal component analysis. SIDER and DICTrank compounds fairly overlap (with the PCA explained variance of 21.47%).

**Figure S2:** Hits from DrugBank compounds containing structural alerts from the top 6 presented in Figure 4(b).


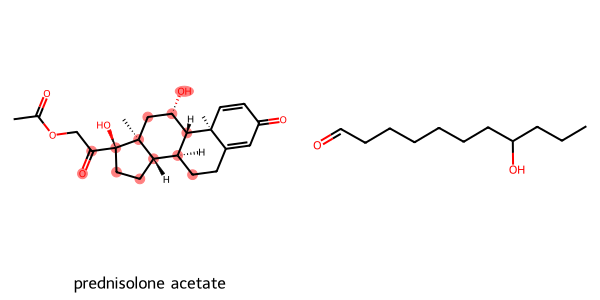


['Cardiac disorders']

Additional Data: ['approved']


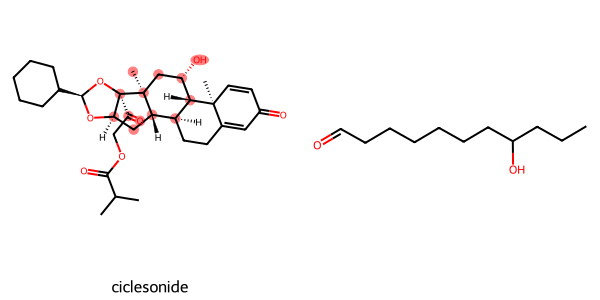


['Cardiac disorders']

Additional Data: ['approved', 'investigational']


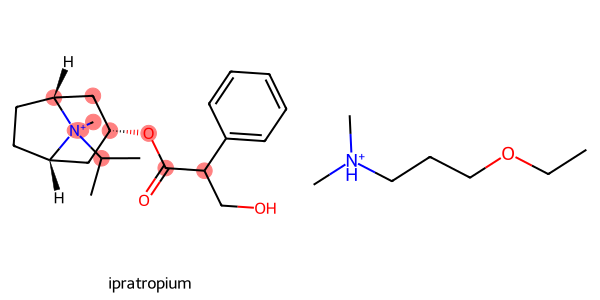


['Cardiac disorders']

Additional Data: ['approved', 'experimental']


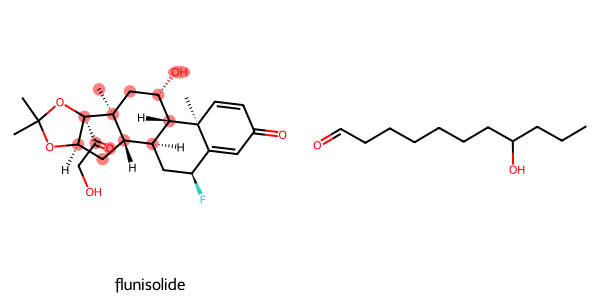


['Cardiac disorders']

Additional Data: ['approved', 'investigational']


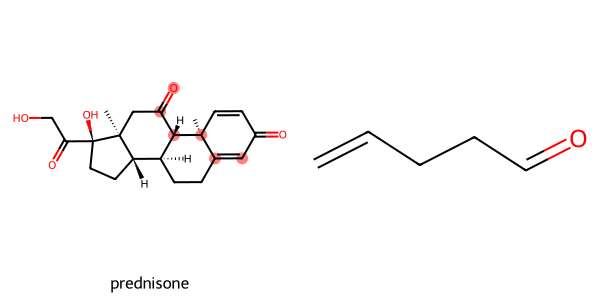


['Cardiac disorders']

Additional Data: ['approved']


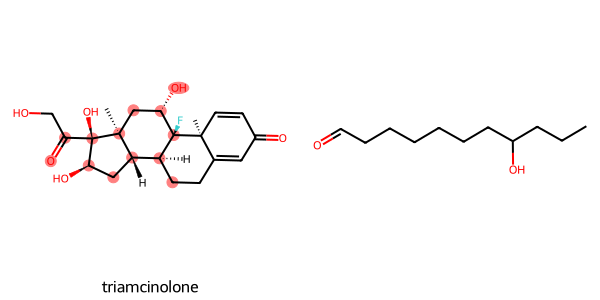


['Cardiac disorders']

Additional Data: ['approved']


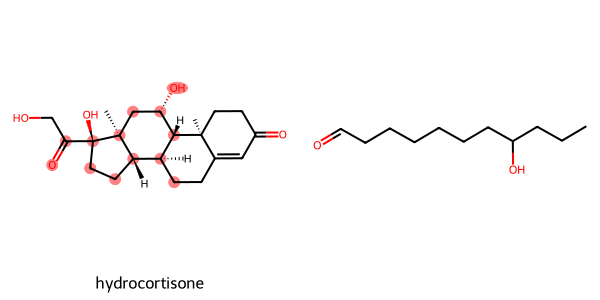


['Cardiac disorders']

Additional Data: ['approved']


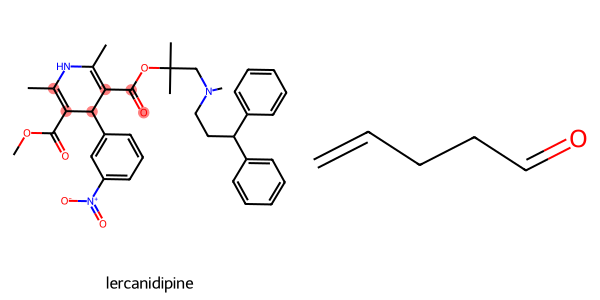


['Cardiac disorders']

Additional Data: ['approved', 'investigational']


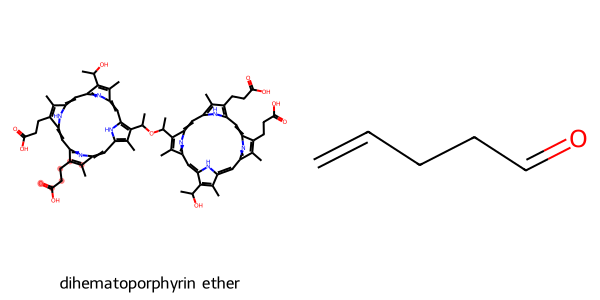


['Cardiac disorders']

Additional Data: ['investigational']


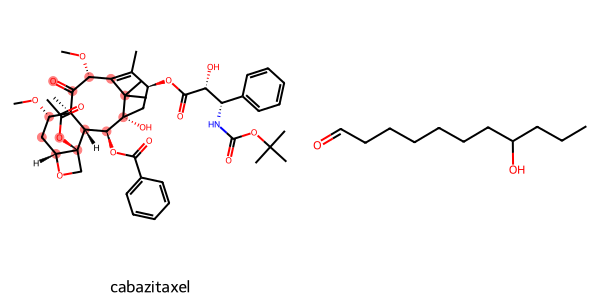


['Cardiac disorders']

Additional Data: ['approved']


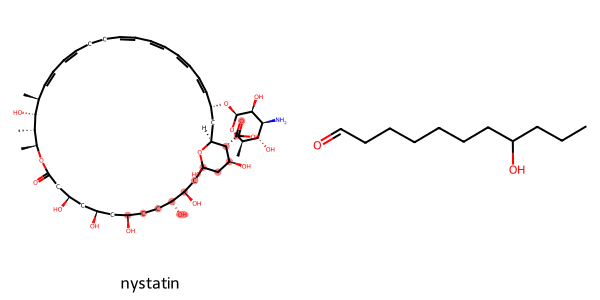


['Cardiac disorders']

Additional Data: ['approved']


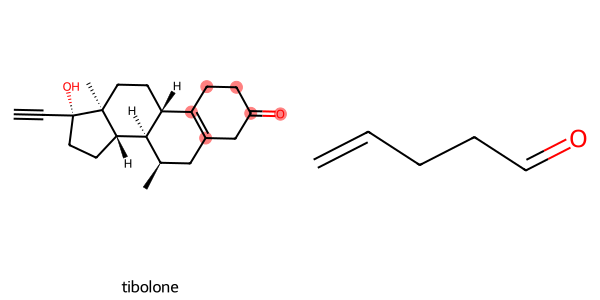


['Cardiac disorders']

Additional Data: ['approved', 'investigational']


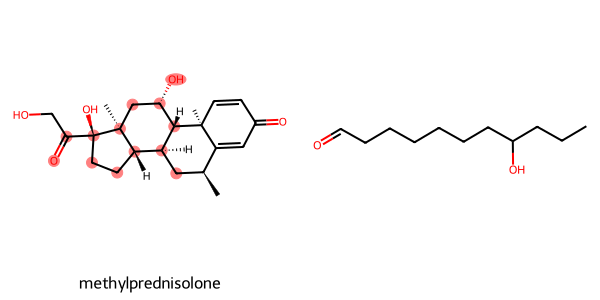


['Cardiac disorders']

Additional Data: ['approved']


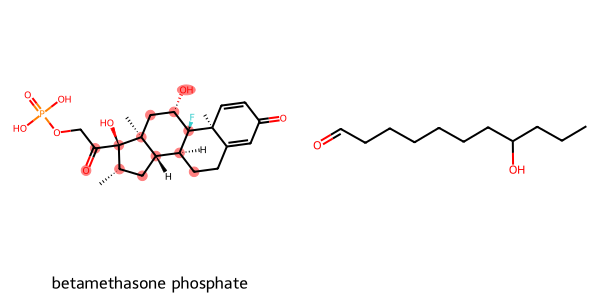


['Cardiac disorders']

Additional Data: ['approved']


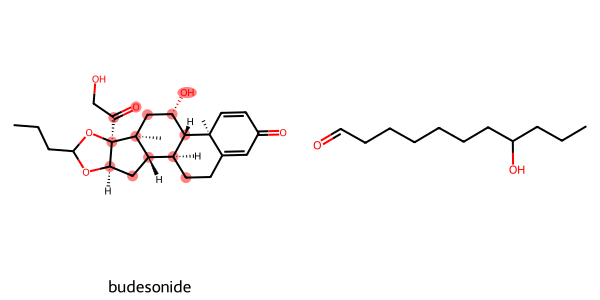


['Cardiac disorders']

Additional Data: ['approved']


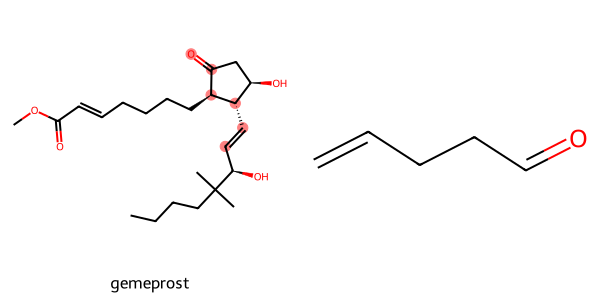


['Cardiac disorders']

Additional Data: ['approved', 'withdrawn']


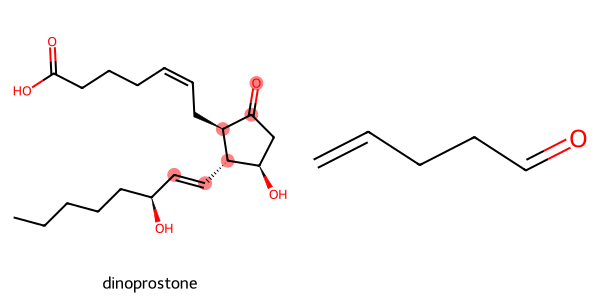


['Cardiac disorders']

Additional Data: ['approved']


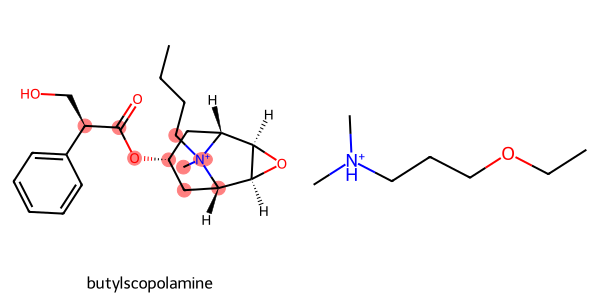


['Cardiac disorders']

Additional Data: ['approved', 'investigational']


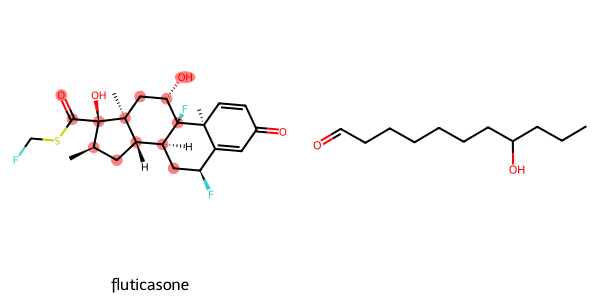


['Cardiac disorders']

Additional Data: ['approved', 'experimental']


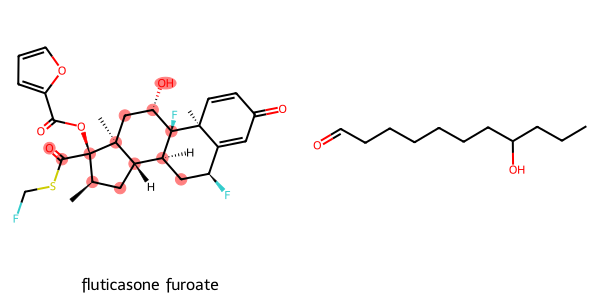


['Cardiac disorders']

Additional Data: ['approved']


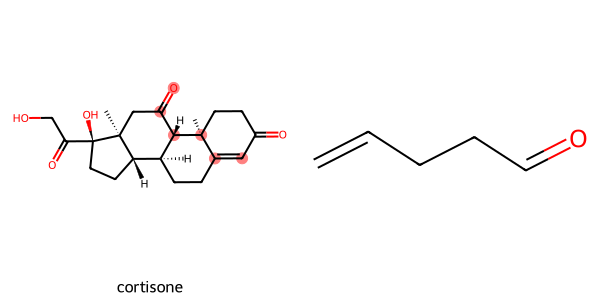


['Cardiac disorders']

Additional Data: ['experimental']


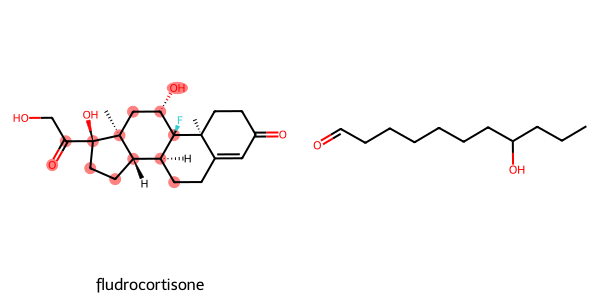


['Cardiac disorders']

Additional Data: ['approved', 'investigational']


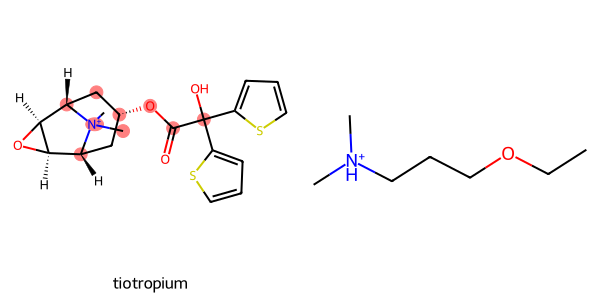


['Cardiac disorders']

Additional Data: ['approved']


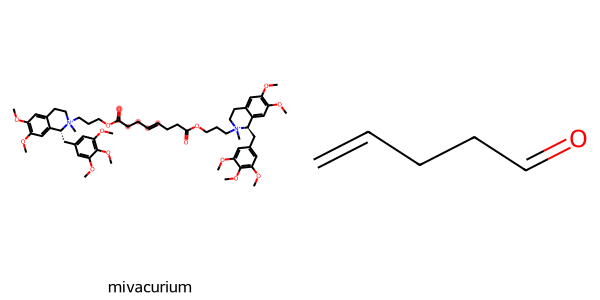


['Cardiac disorders']

Additional Data: ['approved']


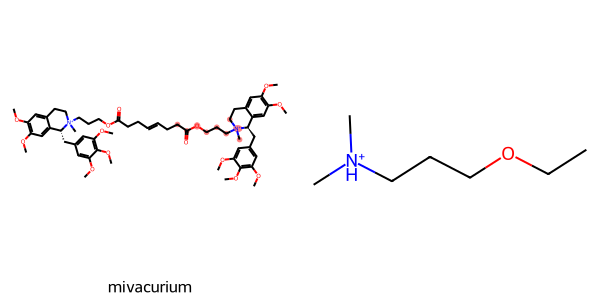


['Cardiac disorders']

Additional Data

: ['approved']


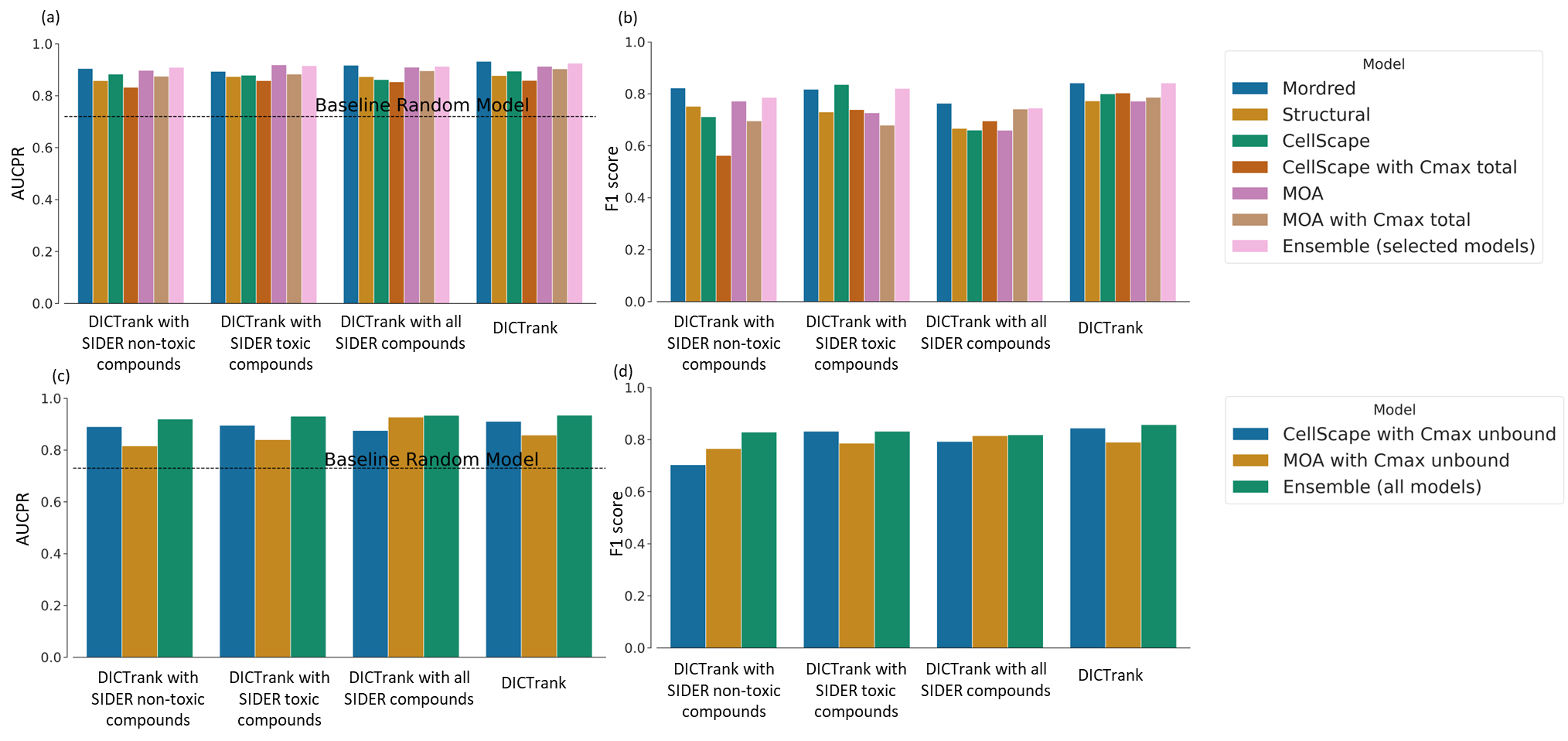


**Figure S3:** Evaluation metrics for models developed in this study using a larger external test set of 90 compounds evaluated using (a) AUCPR and (b) F1 scores, and for models using a subset of the external test set with 78 compounds evaluated using (c) AUCPR and (d) F1 scores
